# Self-reported, functional, and objective health and sociodemographic characteristics among older adults in Kenya: findings from the pilot longitudinal study of health and ageing in Kenya (LOSHAK)

**DOI:** 10.3389/fragi.2025.1693701

**Published:** 2025-12-11

**Authors:** Eunice Muthoni Mwangi, James Orwa, Roselyter Monchari Riang’a, Niranjani Nagarajan, Felix Agoi, Patrick N. Mwangala, Alden L. Gross, Jean N. Ikanga, Kenneth M. Langa, Edward Miguel, Muthoni Gichu, Joshua R. Ehrlich, Anthony K. Ngugi

**Affiliations:** 1 Department of Population Health, Aga Khan University, Nairobi, Kenya; 2 Department of Ophthalmology and Visual Sciences, University of Michigan, Ann Arbor, MI, United States; 3 Environmental Health and Governance Centre, Africa International University, Nairobi, Kenya; 4 Institute for Human Development, Aga Khan University, Nairobi, Kenya; 5 Johns Hopkins University Bloomberg School of Public Health, Baltimore, MD, United States; 6 Johns Hopkins Center on Aging and Health, Baltimore, United States; 7 Department of Rehabilitation Medicine, Atlanta, United States; 8 Department of Psychiatry, University of Kinshasa and Catholic University of Congo, Kinshasa, Democratic Republic of Congo; 9 Department of Internal Medicine and Institute for Social Research, University of Michigan, Ann Arbor, MI, United States; 10 University of Michigan Institute for Social Research, Ann Arbor, MI, United States; 11 University of California Berkeley, Berkeley, CA, United States; 12 Kenya Ministry of Health, Nairobi, Kenya

**Keywords:** older people, aging, self-reported health, functional health, objective health

## Abstract

**Background:**

By 2050, the global population of individuals aged 60 years and older is projected to reach two billion, with 80% residing in low- and middle-income countries (LMICs). Africa’s older population will triple from 74.4 million in 2020 to 235.1 million in 2050, the fastest growth rate globally. Kenya is slightly ahead of the curve on this trajectory, with the population of approximately 2.74 million of the older people expected to quadruple to 12 million over the same period. The Longitudinal Study of Health and Aging in Kenya (LOSHAK) is designed to advance research on population aging in LMICs by focusing on (a) biomarkers and physiological measures; (b) the impacts of air pollution and climate vulnerability; (c) Alzheimer’s disease and related dementias, mental health, disability, caregiving, and psychosocial wellbeing; (d) economic security, including the impact of social welfare; and (e) establish cohorts for long-term study of trajectories of healthy aging and their determinants in a LMIC setting.

**Methods:**

The LOSHAK feasibility and pilot phase was a cross-sectional survey of 203 participants aged 45 years and older. This paper reports on the association between self-reported health and sociodemographic, functional, and objective health measures.

**Results:**

Overall mean age was 63.8 years (SD:11.5) with females accounting for 58.1% (118) of the study population. Based on the wealth index, 111 (54.7%) were classified as poor, with only 75 (36.9%) currently working, with a median income of KShs.11,246.60 (USD 86) over the 3 months preceding the study. Only 32 (15.8%) of respondents reported “very good” self-reported health, while over 80% reported either “somewhat good” 96 (47.3%) or “not good” 75 (36.9%) health status. Multivariable ordinal logistic regression analysis showed that younger age (adjusted odds ratio (aOR): 0.94, 95% CI: 0.91–0.97) and higher subjective wellbeing (aOR: 1.06, 95% CI: 1.02–1.12) were significantly associated with better self-reported health.

**Conclusion:**

This study highlights the importance of considering sociodemographic, subjective wellbeing, and psychosocial factors in improving the health of older adults in Kenya. Including these measures in longitudinal studies of aging and health in Africa in the future is recommended.

## Introduction

Every country in the world is experiencing growth in both the size and the proportion of older adults in the population. By 2050, the proportion of the world’s population over 60 years will nearly double (2.1 billion), and 80% of them will be living in low- and middle-income countries (LMICs) (World Health Organization, 2024). Kenya, like the rest of the LMICs, is experiencing an exceptional demographic transition, with pronounced growth in its older adults, currently estimated at 6% of the country’s total population (Kenya National Bureau of Statistics, 2019). Like many other countries, Kenya is experiencing a growing demand for healthcare services, especially for chronic and age-related conditions, which present major challenges. There is an urgent need to strengthen the health and social systems to respond to this demographic shift effectively (World Health Organization, 2024). Thus, it is important to measure the wellbeing of older people and to build a system capable of responding to their individual needs and experiences (Fukui et al., 2021).

Several measures have been used in assessing older adults’ health and wellbeing (Hackert et al., 2021; Hochwälder et al., 2022; Nainee et al., 2023). A well-known example is HelpAge International’s Global Age Watch Index, developed in 2013, which measures the wellbeing of older people based on key priorities related to income security, education, employment, an enabling environment, self-reported health status, and psychological wellbeing. Self-reported health is a single-item subjective measure where individuals evaluate their own health status on a scale, typically from excellent to poor (Wu et al., 2013). Self-reported health has proven remarkably effective in outcome measures in public health (Falk et al., 2017) and predicting health outcomes such as morbidity, disability, and mortality (Wu et al., 2013; Wuorela et al., 2020; DeSalvo et al., 2006; Idler and Benyamini, 1997) in addition to evaluating health interventions in resource-limited settings (Falk et al., 2017). It is often correlated with objective and functional health status and can serve as a global measure of health status in the general population (Wu et al., 2013). While self-reported health is a widely used and valid global indicator of health status across populations, interpretation should always be made in light of the specific demographic group being studied. Objective health is assessed through quantifiable indicators of an individual’s health, such as clinical evaluations, diagnostic tests, and medical examinations, which provide an empirical assessment of a person’s health condition, independent of their subjective perceptions or self-reporting. Functional health evaluates an individual’s ability to perform activities, such as Activities of Daily Living (ADLs), independently (Frangos et al., 2023; Tóth et al., 2025; Menassa et al., 2023).

Kenya lacks national representative data for older people, and little is known about self-reported health in LMICs; it is not clear which objective, functional, and health measures are associated with it. The study, therefore, aims to bridge the knowledge gap by exploring how sociodemographic, subjective perceptions, clinical data, and functional abilities correlate and influence self-reported health outcomes using data from the recently concluded pilot and feasibility phase of the Longitudinal Study of Health and Aging in Kenya (LOSHAK).

## Materials and methods

### Study design and sampling

The LOSHAK feasibility and pilot phase was a cross-sectional survey of a sample of 203 participants aged ≥45 years purposively selected from the Kaloleni/Rabai Community Health and Demographic Surveillance System (KRHDSS) (Ngugi et al., 2020). Subsequent full population-representative waves of LOSHAK will adopt a longitudinal panel survey design. KRHDSS operates in Kaloleni and Rabai sub-counties, two of seven sub-counties of Kilifi County in the coastal region of Kenya and is mapped onto 10 community health units (CHUs). Four CHUs were purposively selected, with two in rural and the other two in urban/semi-urban regions. One rural and one urban/semi-urban CHU were drawn from each of the two sub-counties. The participants were sampled proportionate to the population distribution by age group (45–54, 55–64, and 65+ years) and sex (male/female) within each sub-county. The sampling approach used was to facilitate comparisons of measures between rural and urban/peri-urban settings, between age groups, and by sex (See Table 1). Recruitment was conducted in two stages. In the first stage, the LOSHAK team visited communities to build rapport with key stakeholders, including community leaders, local administrators and community health workers, to provide study information and address questions. In the second stage, interviewers contacted pre-identified participants to schedule and conduct face-to-face interviews, explained the study, and obtained informed consent, including from proxy informants when participants were unable to respond directly. For illiterate participants, consent forms were read aloud, and thumbprints were used in the presence of a witness. The field team, comprising a study coordinator, four interviewers, a field supervisor, and a quality assurance officer, underwent 2 weeks of intensive training on study procedures, data collection, quality assurance and participant engagement. All instruments and protocols were pretested and refined to ensure data quality and interviewer readiness (Nagarajan et al., 2024).

**TABLE 1:** Target population and sample size distribution.

Sub-county	Community health unit	Age group (Years)			
		45–54 Total (sampled)	55–64 Total (sampled)	65+ Total (sampled)	Total 45+ Total (sampled)
Kaloleni	Vishakani (Urban/Peri-urban)	1,666 (28)	998 (17)	892 (15)	3,556 (60)
	Kwale (Rural)	419 (24)	320 (18)	313 (18)	1,052 (60)
Rabai	Mwele-Kisurutini (Urban/Peri-urban)	937 (27)	562 (16)	609 (17)	2,108 (60)
	Kombeni (Rural)	718 (26)	478 (17)	457 (17)	1,653 (60)
Total (sample size)	​	3,740 (105)	2,358 (68)	2,271 (67)	8,369 (240)

### Ethical considerations

Ethical clearance was granted by the Institutional Scientific and Ethical Review Committees (ISERC) at Aga Khan University (AKU) (approval number 2022/ISERC—109 (v3) and the University of Michigan (HUM00224039). Research permits and approvals were acquired from the Kilifi County Department of Health (KLF/DOH/RESEARCH/Vol 1/021), and licensing was obtained from the Kenyan research regulatory body, the National Council of Science and Technology (NACOSTI/P/23/24193), before commencement. Participation in the project was voluntary, with individuals providing informed consent. Respondents were encouraged to ask questions before consenting and had the freedom to withdraw from the study at any point. To safeguard participant confidentiality, unique identifiers were utilized for participant and household identification. All data were securely transferred to an encrypted, password-protected online database. Access to the data was restricted to the Principal Investigator (PI) and the research team.

### Measurements and data collection tools

Four trained interviewers administered the questionnaires adapted from the Health and Retirement Study (HRS) network of sister studies, particularly from the Longitudinal Aging Study in India (LASI) and Health and Aging in Africa: A Longitudinal Study of an INDEPTH Community (HAALSI) (Riang’a et al., 2025). The questionnaires were translated from English to Swahili and contextualized to the local context by a team of local researchers before adoption (Riang’a et al., 2025). To ensure consistency and preservation of meaning during translation, the instruments were first translated from English to Swahili by a professional translator fluent in both languages and familiar with public health terminologies. A separate, independent translator, who was blinded to the original English version, then back-translated the Swahili version into English. The two English versions were compared by the study team to identify and resolve discrepancies through consensus discussions, focusing on conceptual rather than literal equivalence. The finalized Swahili version was further pretested with a small sample of respondents from the target population to confirm clarity, cultural appropriateness, and comprehension before electronic programming in ODK. This process followed WHO standards to ensure rigorous translation (Ozolins et al., 2020). Data transmission in ODK was secured through end-to-end encryption, ensuring that responses were safely transferred from mobile devices to the server. Participants were given unique codes, and access to de-identifiable information was restricted by using password-protected accounts and allowing only authorized research personnel to view or manage data. The LOSHAK pilot survey included assessments of various variables (Supplement 1) (Nagarajan et al., 2024); however, this paper reports on the sociodemographic characteristics, self-reported health, functional and objective health measures, as presented in Table 2.

**TABLE 2:** Sociodemographic, self-reported, functional, and objective health Measures.

Topic area	Measures	Domain interpretation
Sociodemographic	Age, gender, wealth index, level of education, marital status, occupation, income last 3 months, social welfare program, electricity in the household, livestock owned, number of sleeping rooms	The household wealth index was calculated based on the assets owned by household members and their housing characteristics, using principal component analysis, and categorized into five quintiles (Rutstein, 2015). Due to the small sample size, the first and second quantiles were combined into the ‘poor’ category, and the fourth and fifth quantiles were combined into the “rich” category
Self-reported health	Self-reported health was measured by a single question. “Would you describe your general health as very good, somewhat good, or not good?”	Response options were 1 = not good, 2 = somewhat good, and 3 = very good
Functional health (psychosocial, mental health and behavioral)	Radloff’s CES-D (10-item) (Radloff, 1977)	Depressive symptoms: Scored from 0 (Rarely or none of the time) to 3 (All the time), except for items 5 and 8, where scores were reversed. The total score ranging from 0 to 30 was used to assess depressive symptoms, with a score of 0–4 indicating no depressive symptoms, 5–9 mild depressive symptoms, 10–14 moderate depressive symptoms, and 15–30 severe depressive symptoms (Baron et al., 2017)
	3-Item loneliness scale	Loneliness: Measured using a 3-item scale (Hughes et al., 2004), with items initially scored from 1 (Often) to 3 (Hardly ever or never). These scores were then reverse coded, with 3 indicating “Often,” 2 for “Some of the time,” and 1 for “Hardly ever or never.” The scores were summed to yield an overall loneliness score of nine, dichotomized into 3–5 (not lonely) and 6–9 (lonely)
	CASP-19	Subjective wellbeing: The CASP-19 scale (Hyde et al., 2003) assessed subjective wellbeing across 19 Likert-type items, where participants rated statements like “I feel left out of things” on a four-point scale from 0 (Never) to 3 (Often). Items 1, 2, 4, 6, 8, and 9 were negatively worded and reverse coded. Total scores ranged from a minimum of 0 (complete lack of quality of life) to a maximum of an absolute score of 57 (complete satisfaction with control, autonomy, self-realization, and pleasure)
	Ill treatment	Ill-treatment (International Institute for Population Sciences, 2020) was categorized into yes or no responses, and if yes, whether it occurred at home, outside, or both
	Single-item life satisfaction scale	Life satisfaction was assessed by a single question: “In general, how satisfied are you with your life?” on a 4-point scale ranging from 1 (Very Satisfied) to 4 (Very Dissatisfied) (Lucas and Brent, 2012). Values were reverse-coded, with higher values representing greater life satisfaction
	MacArthur ladder	Subjective social status: The MacArthur Ladder of Subjective Social Status (Adler et al., 2000) score was categorized into three levels: High (Falk et al., 2017; Wuorela et al., 2020; DeSalvo et al., 2006), Middle (Hackert et al., 2021; Hochwälder et al., 2022; Nainee et al., 2023; Wu et al., 2013), and Low (World Health Organization, 2024; Kenya National Bureau of Statistics, 2019; Fukui et al., 2021) subjective social status
	4-Item perceived stress scale	Stress: The 4-item perceived stress scale had scores for positive items 2 and 3 reversed (0 = 4, 1 = 3, 2 = 2, 3 = 1, 4 = 0) (Cohen et al., 1983). The total score ranged from 0 to 16, with higher scores indicating more perceived stress. Stress levels were categorized as follows: Low Stress (<6), Moderate Stress (6–9), and High Stress (10 or above)
	Single-item financial strain	Financial strain was assessed using Kahn and Pearlin, (2006) framework (Kahn and Pearlin, 2006)
​	Disability was assessed across multiple domains using the washington group short set	Disability was categorized into four levels: (Disability1: Difficulty in at least one domain), (Disability2: Difficulty in at least two domains or significant difficulty in one domain), (Disability3: Severe difficulty in any one domain), and (Disability4: Inability to perform at least one domain – cannot do at all) (Washington Group on Disability Statistics, 2022) However, due to the small number of those with disabilities, this was further classified into those with and without disability
Objective health (physiological/anthropometric)	Blood pressure and pulse were measured using an Omron HEM-780N Monitor. A cuff was secured around the participant’s left upper arm. The participant was seated comfortably with legs uncrossed, feet flat on the floor, and the arm supported at heart level, palm facing upward. Three consecutive measurements were taken at 1-min intervals without removing the cuff between readings. An average of the last two readings was recorded as the final score	Blood pressure was classified following the Kenya National Guidelines For Cardiovascular Diseases Management (Ministry of Health, 2018) however, due to the study small sample size, the seven categories in the guidelines were reclassified to five categories: Normal (Systolic BP (SBP) < 120 and Diastolic BP (DBP) < 80), Elevated/Pre-Hypertension (SBP ≤120 and DBP <80), Stage 1 Hypertension (SBP between 130 and 139 or DBP between 80 and 89), Stage 2 Hypertension (SBP ≥140 or DBP ≥90), and Hypertensive Crisis (SBP ≥180 or DBP ≥120) (Whelton et al., 2017). Respondents found to have stage 2 and in hypertensive crisis were referred to the closest health facility
	Grip strength	Hand grip strength was measured using a standard adjustable digital hand grip dynamometer, with participants asked to exert maximum force three times with each hand. Results are based on the average of three trials using the dominant hand, recorded in kilograms (Roberts et al., 2011)
	Height/Weight Height was measured using a stadiometer. The participant removed their shoes and stood upright on the base of the device with feet together, knees straight, heels, buttocks, and back against the wall. The head was positioned with the chin slightly tucked and eyes looking straight ahead. The measurement was read in centimeters and recorded to the nearest 0.1 cm Weight was measured in kg using a calibrated weighing scale. The participant removed shoes and any bulky clothing before standing still on the scale, looking straight ahead	The Body Mass Index (BMI) categories used were defined as per standard definitions as follows: underweight, BMI <18.5, normal BMI 18.5 to <25, overweight BMI 25 to <30, and obese ≥30 BMI (World Health Organization, 2000)
	Waist/Hip circumference Waist and hip circumferences were measured using a soft tape measure placed at the level of the navel and the widest part of the hips, respectively. For both measurements, the participant stood upright, and the measurement was taken after a normal exhalation, with the participant holding their breath briefly at the end of the exhale. Measurements were recorded to the nearest 0.1 cm	A Waist-to-Hip Ratio (WHR) greater than 0.85 (women) and 1.0 (men) is considered a high risk for cardiovascular disease and other health complications (World Health Organization, 2008)

### Data management and analysis

Data were analyzed descriptively using frequency and percentages for categorical outcomes and mean (standard deviation) or median (interquartile range) for continuous variables. Chi-square or Fisher’s exact test was used to compare categorical variables across different categories of Self-reported health, whilst continuous variables were compared using the Kruskal–Wallis test. Any variable with a p-value <0.2 in the bivariable analysis or considered clinically relevant in explaining Self-reported health was adjusted for in the multivariable ordinal logistic regression model. All analyses were performed using R software version 4.3.2 (2023-10-31 ucrt) and p-value <0.05 was set as the level of statistical significance.

## Results

### Sociodemographic characteristics and self-reported health

Self-reported health and its association with sociodemographic characteristics are shown in Table 3. Among the 203 participants, the overall mean age in years was 63.8 (SD: 11.5), 118 (58.1%) of the sample were females, 104 (51.2%) had no formal education, 111 (54.7%) were classified in the poor wealth index category, 75 (36.9%) were currently working, while 35 (17.2%) of households had at least one member in a social welfare program. Only 32 (15.8%) participants rated their health as “very good,” while nearly half reported “somewhat good” (96; 47.3%), and 75 (36.9%) described their health as “not good.” Worth noting is that women comprised the majority of those reporting poorer health, accounting for 55 (57.3%) of the “somewhat good” and 49 (65.3%) of the “not good” self-reported health groups. Age, employment, and involvement in social welfare programs were significantly associated with self-reported health. A statistically significant difference in the mean age of the respondents across different self-reported health categories (p < 0.001) was observed. Older participants tended to report poorer health, as shown by the statistically significant difference in mean age across self-reported health categories. Being currently employed was linked to better self-reported health (p < 0.001), suggesting that employment may be associated with improved wellbeing or access to resources. In addition, involvement in social welfare programs such as monthly pension payment for (60+ years), relief food support, cash transfer for elderly persons (70+ years), cash transfer for persons with disability, orphans and vulnerable children, including widowers, widows, and HIV was associated with lower self-reported health (p-value = 0.036). Only 35 (17.2) reported receiving social support. Respondents reporting “not good” health 19 (25.3%) were more likely to be part of a social welfare program compared to those in “very good” health 2 (6.3%), and those in “somewhat good” health, 14 (14.6%), implying that social welfare support may be reaching those with greater health or socioeconomic vulnerabilities.

**TABLE 3:** Sociodemographic characteristics and their association with self-reported health among older adults in Kilifi County, Kenya.

Characteristics	Overall, n = 203	Self-reported health			p-value^a^
		Very good, n = 32	Somewhat good, n = 96	Not good, n = 75	
Respondent’s age (years), Mean (SD)	63.8 (11.5)	57.1 (8.9)	62.8 (11.3)	67.9 (11.2)	<0.001
Sex, n (%)	​	​	​	​	0.11
Male	85 (41.9)	18 (56.3)	41 (42.7)	26 (34.7)	
Female	118 (58.1)	14 (43.8)	55 (57.3)	49 (65.3)	
Wealth index, n (%)	​	​	​	​	0.35
Poor	111 (54.7)	22 (68.8)	46 (47.9)	43 (57.3)	
Middle	63 (31.0)	7 (21.9)	35 (36.5)	21 (28.0)	
Rich	29 (14.3)	3 (9.4)	15 (15.6)	11 (14.7)	
Highest level of education, n (%)	​	​	​	​	0.019
Non-formal	104 (51.2)	14 (43.7)	42 (43.8)	48 (64.0)	
Primary	74 (36.5)	15 (46.9)	36 (37.5)	23 (30.7)	
Secondary	25 (12.3)	3 (9.4)	18 (18.8)	4 (5.3)	
Marital status, n (%)	​	​	​	​	0.39
Divorced	6 (3.0)	1 (3.1)	4 (4.2)	1 (1.4)	
Married	133 (66.5)	22 (68.8)	67 (70.5)	44 (60.3)	
Missing	21 (10.5)	2 (6.3)	7 (7.4)	12 (16.4)	
Single	5 (2.5)	2 (6.3)	2 (2.1)	1 (1.4)	
Widow	35 (17.5)	5 (15.6)	15 (15.8)	15 (20.5)	
HH occupation, n (%)	​	​	​	​	0.067
Employed	29 (15.3)	5 (16.1)	15 (16.3)	9 (13.4)	
Casual labourer	38 (20.0)	13 (41.9)	15 (16.3)	10 (14.9)	
Business	22 (11.6)	3 (9.7)	12 (13.0)	7 (10.4)	
Farmer/Other	101 (53.2)	10 (32.3)	50 (54.3)	41 (61.2)	
Spouse occupation, n (%)	​	​	​	​	0.26
Employed/Casual labourer/Business	24 (12.6)	4 (12.9)	14 (15.2)	6 (9.0)	
Farmer	84 (44.2)	9 (29.0)	44 (47.8)	31 (46.3)	
House wife	44 (23.2)	9 (29.0)	16 (17.4)	19 (28.4)	
Other/Not applicable	38 (20.0)	9 (29.0)	18 (19.6)	11 (16.4)	
Currently working, n (%)	75 (36.9)	19 (59.4)	40 (41.7)	16 (21.3)	<0.001
Total income last 3 months, Mean (SD) (KES)	11,246.6 (21,421.4)	16,694.9 (31,067.5)	10,250.0 (18,431.7)	7,233.3 (12,899.2)	0.84
HH member part of a social welfare program, n (%)	35 (17.2)	2 (6.3)	14 (14.6)	19 (25.3)	0.036
Has electricity in the HH, n (%)	91 (44.8)	13 (40.6)	47 (49.0)	31 (41.3)	0.53
Owns livestock, n (%)	155 (76.4)	23 (71.9)	73 (76.0)	59 (78.7)	0.75
Number of rooms sleeping rooms in HH, Mean (SD)	2.6 (1.0)	2.5 (0.9)	2.8 (1.0)	2.5 (0.9)	0.12

^a^ Kruskal–Wallis rank sum test; Pearson’s Chi-squared test; Fisher’s exact test.

Sex, marital status, occupations, household assets, total income over the previous 3 months, access to electricity, livestock ownership, and number of sleeping rooms were not statistically significantly associated with self-reported health.

### Functional health and self-reported health

Self-reported health and its association with functional health are shown in Table 4. Statistically significant associations were found between self-reported health and several functional health indicators. Subjective wellbeing varied across self-reported health categories (p < 0.001), with higher wellbeing scores among participants reporting “very good” health (mean score 54.8, SD: 9.6). This shows that individuals who viewed their health more positively also reported better overall health. Perceived stress also differed significantly (p < 0.001), with a mean of 6.3 among those with “very good” health and 8.1 among those with “not good” health, indicating that poorer perceived health was linked to higher stress levels. Disability status (difficult seeing, hearing, walking or climbing steps, remembering or concentrating, difficult with self-care such as washing all over or dressing, and difficulty communicating) varied significantly across self-reported health categories (p = 0.007), with greater severity among those perceiving their health as worse, underscoring the connection between functional limitations and lower perceived health. Perceived social status had a statistically significant relationship with self-reported health (p = 0.005), as most participants (83.5%) rated themselves in the low or middle categories. A strong desire for higher social status was also significantly associated with self-reported health (p = 0.031), being more common among those reporting better perceived health. This suggests that individuals who felt healthier were also more optimistic or motivated about improving their social position.

**TABLE 4:** Association between self-reported and functional health among older adults in Kilifi County, Kenya.

Characteristics	Overall, N = 203	Self-reported health			p-value^a^
		Very good, N = 32	Somewhat good, N = 96	Not good, N = 75	
Depression score, Mean (SD)	17.4 (6.1)	16.6 (4.6)	17.4 (5.7)	17.8 (7.0)	0.64
Depressive symptoms, n (%)	​	​	​	​	0.005
No depression	12 (5.9)	1 (3.1)	4 (4.2)	7 (9.3)	
Mild	43 (21.2)	13 (40.6)	24 (25.0)	6 (8.0)	
Moderate	70 (34.5)	7 (21.9)	33 (34.4)	30 (40.0)	
Severe	78 (38.4)	11 (34.4)	35 (36.5)	32 (42.7)	
Loneliness, n (%)	​	​	​	​	0.12
Lonely (Score 6–9)	55 (27.1)	4 (12.5)	30 (31.3)	21 (28.0)	
Not lonely (Score 3–5)	148 (72.9)	28 (87.5)	66 (68.8)	54 (72.0)	
Subject wellbeing score, Mean (SD)	54.8 (9.6)	62.9 (8.4)	55.3 (7.7)	50.6 (10.1)	<0.001
Felt ill-treated in the past year, n (%)	90 (44.3)	15 (46.9)	47 (49.0)	28 (37.3)	0.30
Place of ill-treatment, n (%)	​	​	​	​	0.33
At home	31 (34.4)	5 (33.3)	20 (42.6)	6 (21.4)	
Outside	55 (61.1)	10 (66.7)	25 (53.2)	20 (71.4)	
Both	4 (4.4)	0 (0.0)	2 (4.3)	2 (7.1)	
Life satisfaction score, Mean (SD)	18.0 (8.6)	17.7 (9.9)	18.7 (8.5)	17.4 (8.3)	0.60
Life satisfaction, n (%)	​	​	​	​	0.35
Satisfied	54 (26.6)	10 (31.3)	25 (26.0)	19 (25.3)	
Slightly satisfied	40 (19.7)	3 (9.4)	24 (25.0)	13 (17.3)	
Dissatisfied	109 (53.7)	19 (59.4)	47 (49.0)	43 (57.3)	
Perceived stress score, Mean (SD)	7.6 (1.9)	6.3 (2.5)	7.7 (1.8)	8.1 (1.4)	<0.001
Financial strain, n (%)	​	​	​	​	0.13
Very hard	68 (33.5)	9 (28.1)	27 (28.1)	32 (42.7)	
Hard	57 (28.1)	7 (21.9)	27 (28.1)	23 (30.7)	
Somewhat hard	53 (26.1)	10 (31.3)	31 (32.3)	12 (16.0)	
Not very hard	25 (12.3)	6 (18.8)	11 (11.5)	8 (10.7)	
Disability scores, Mean (SD)	7.3 (1.7)	6.5 (0.7)	6.9 (1.1)	8.2 (2.2)	<0.001
Disability status, n (%)	​	​	​	​	0.007
Without disability	187 (92.1)	31 (96.9)	93 (96.9)	63 (84.0)	
With disability	16 (7.9)	1 (3.1)	3 (3.1)	12 (16.0)	
Where you place yourself, n (%)	​	​	​	​	0.005
Low	62 (32.0)	4 (14.3)	34 (35.4)	24 (34.3)	
Middle	100 (51.5)	12 (42.9)	50 (52.1)	38 (54.3)	
High	32 (16.5)	12 (42.9)	12 (12.5)	8 (11.4)	
Place you would like to achieve in your life, n (%)	​	​	​	​	0.031
Low	1 (0.5)	0 (0.0)	1 (1.0)	0 (0.0)	
Middle	34 (17.4)	5 (17.2)	10 (10.4)	19 (27.1)	
High	160 (82.1)	24 (82.8)	85 (88.5)	51 (72.9)	

^a^ One-way ANOVA; Fisher’s exact test; Pearson’s Chi-squared test; Kruskal–Wallis rank sum test.

No statistically significant associations were found between self-reported health and depressive symptoms score, loneliness, ill-treatment, life satisfaction, or financial strain. Depressive symptoms were reported by 191 participants (94.1%), with 21.2%, 34.5%, and 38.4% experiencing mild, moderate, and severe symptoms, respectively. The mean score (17.4, SD: 6.1) rose from 16.6 among those reporting “very good” health to 17.8 for those reporting “not good” health, though the difference was not significant (p = 0.64). Loneliness was reported by 27.1% of participants, mainly among those with “somewhat” or “not good” health, though this difference was not statistically significant (p = 0.12). Perceived ill-treatment was reported by 90 (44.3%) of the participants, mostly occurring outside the home (61.1%), with no significant variation across self-reported health categories (p = 0.33). Life satisfaction was low overall, with a mean score of 18.0(SD: 8.6); over half 109 (53.7%) reported dissatisfaction with life based on this scale, although no significant difference across self-reported health categories was noted (p = 0.35). Although over 60% (125) reported financial strain, there was no statistically significant association between financial strain and self-reported health (p = 0.13).

### Objective health and self-reported health

Results of self-reported health and its association with objective health are presented in Table 5. Statistically significant associations were observed between self-reported health and male hand grip strength, and male and female blood pressure. Among males, the median hand grip strength decreased markedly from 35.4 kg (IQR: 30.1–44.0) among those reporting “very good” health to 23.4 kg (IQR: 19.1–28.5) among those reporting “not good” health (p < 0.001), indicating that better self-reported health was associated with greater physical strength. Blood pressure readings also showed a statistically significant relationship with self-reported health (p = 0.04). Most participants, 112 (56.3%), were classified in stage 2 or the hypertension crisis group, with over 90% of these reporting “somewhat good” to “not good” health status. Those identified as being at risk of hypertension were referred to the nearest public health facility.

**TABLE 5:** Association between self-reported and objective health status among older adults in Kilifi County, Kenya.

Characteristics	Overall, N = 203	Self-reported health			p-value^a^
		Very good, N = 32	Somewhat good, N = 96	Not good, N = 75	
Hand grip strength scores: Female, Median (IQR)	20.5 (17.5–25.2)	22.3 (18.5–25.5)	20.8 (18.2–25.8)	19.5 (16.3–3.5)	0.21
Hand grip strength scores: Male, Median (IQR)	29.1 (22.8–34.9)	35.4 (30.1–44.0)	29.2 (24.6–33.9)	23.4 (19.1–28.5)	<0.001
BMI (kg/m2), Median (IQR)	20.8 (18.1–24.5)	20.8 (18.4–3.7)	21.2 (18.3–4.6)	20.5 (18.0–4.8)	0.73
Categories of BMI, n (%)	​	​	​	​	0.86
Underweight	55 (28.1)	9 (30.0)	24 (25.3)	22 (31.0)	
Normal	98 (50.0)	16 (53.3)	48 (50.5)	34 (47.9)	
Overweight and Obese	43 (21.9)	5 (16.7)	23 (24.2)	15 (21.1)	
Blood pressure, n (%)	​	​	​	​	0.040
Normal	26 (13.1)	6 (20.0)	14 (14.6)	6 (8.2)	
Elevated	16 (8.0)	3 (10.0)	6 (6.3)	7 (9.6)	
Stage 1	45 (22.6)	12 (40.0)	19 (19.8)	14 (19.2)	
Stage 2/Hypertensive crisis	112 (56.3)	9 (30.0)	57 (59.4)	46 (63.0)	
Health risk based on waist hip ratio: Female, n (%)	​	​	​	​	0.21
At high risk	84 (41.6)	10 (31.3)	38 (39.6)	36 (48.6)	
Not at risk	118 (58.4)	22 (68.8)	58 (60.4)	38 (51.4)	
Health risk based on waist hip ratio: Male, n (%)	​	​	​	​	0.44
At high risk	33 (16.3)	6 (19.4)	18 (18.8)	9 (12.0)	
Not at risk	169 (83.7)	25 (80.6)	78 (81.3)	66 (88.0)	

^a^ Kruskal–Wallis rank sum test; Pearson’s Chi-squared test; Fisher’s exact test.

**FIGURE 1:**
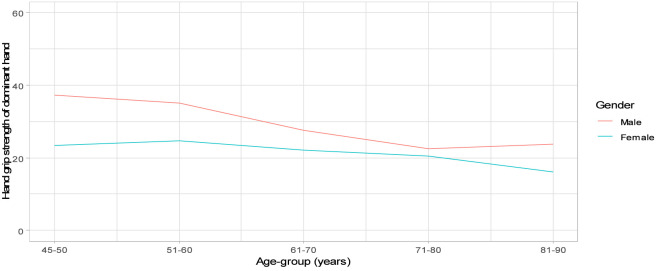
Handgrip strength by gender and age group.

No statistically significant associations were observed between self-reported health and female hand grip strength. Among women, the median hand grip strength was 22.3 kg (IQR: 18.5–25.5) for those with “very good” health and 19.5 kg (IQR: 16.3–23.5) for those with “not good” health (p = 0.21). The distribution of dominant hand grip strength by age showed that males had higher grip strength than females, with a steady decline in males as age increased. In females, grip strength remained relatively stable until ages 81–90 years, after which it declined more sharply. The distribution of dominant hand grip for males and females by age is shown in Figure 1. Body Mass Index (BMI) did not differ significantly across self-reported health categories (p = 0.73); about half of the participants had a healthy weight. Additionally, based on waist-to-hip ratio, the majority 117 (57.6%) were classified as being in the high health risk category.

**FIGURE 2:**
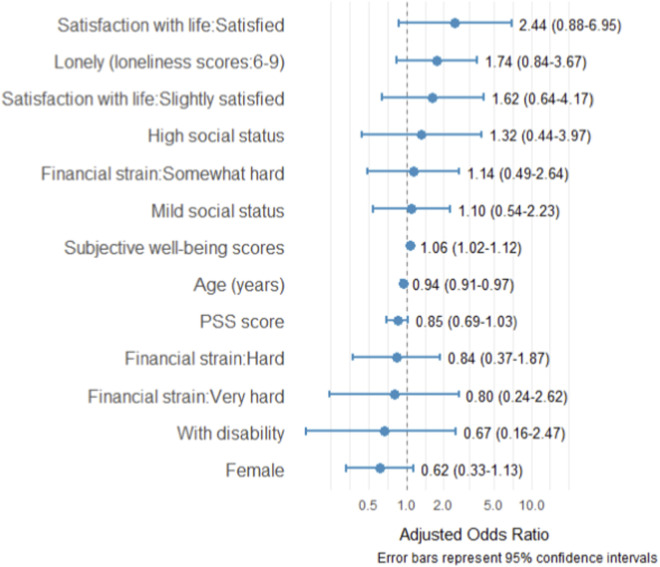
Ordinal logistic regression parameters estimate of the risk factors associated with self-reported health among older adults in Kilifi County, Kenya. PSS: Perceived stress scores.

The study’s multivariable ordinal logistic regression analysis, as presented in Figure 2, revealed that older age, which is one of the sociodemographic predictors, was statistically significantly associated with lower self-reported health (adjusted odds ratio [aOR], 0.94; 95% CI, 0.91–0.97; P < 0.001). Similarly, a higher subjective wellbeing score, which is a functional covariate, was associated with greater odds of reporting better self-reported health (aOR, 1.06; 95% CI, 1.02–1.12; P = 0.002). Other variables (satisfaction with life, loneliness, perceived social status, financial strain, perceived stress, sex, and disability) were not statistically significantly related to self-reported health in the multivariable model.

## Discussion

Sociodemographic characteristics, functional health, and objective health were assessed for their association with self-reported health categories: “very good,” “somewhat good,” and “not good.” Among the 203 participants in LOSHAK’s pilot phase, only 32 (15.8%) reported their health as “very good”. Women comprised the majority among those reporting poorer health. A comprehensive WHO study conducted across six countries established that self-reported health is a valid and reliable indicator of health status in LMICs, providing a simple yet powerful measure of population wellbeing (Falk et al., 2017). Among older adults in LMICs, self-reported health is shaped by a complex interplay of socioeconomic, psychological, and physical factors. The prevalence of poor self-reported health varies considerably across settings, ranging from 11.6% to 56.4%, reflecting substantial cross-country differences. For instance, one study reported that 11.6% of older adults across six LMICs rated their health as poor (Arokiasamy et al., 2015), while another found a considerably higher prevalence of 56.4% in Togo (Gbeasor-Komlanvi et al., 2020), particularly among women and those aged over 60. In Malaysia, a separate study observed a prevalence of 32.6% among older persons (Sahril et al., 2023).

Across LMICs, older men consistently report better self-rated health than older women. Large multi-country studies attribute these sex differences to variations in demographic and socioeconomic factors across cultural contexts (Selv et al.), social capital, including access to bridging networks and personal trust, which have been found to have a positive impact on perceived health (Ng et al., 2015). Physical health factors such as chronic diseases, functional limitations, and reduced physical capacity remain central determinants (Arnadottir et al., 2011), while psychological wellbeing, particularly depression, exerts the strongest negative influence on self-reported health (Falk et al., 2017). Consistent demographic patterns further reveal that men tend to report better health than women across diverse national contexts (Ng et al., 2015). Collectively, these findings underscore the importance of understanding contextual factors when assessing the health and wellbeing of older populations in LMICs.

Overall, sociodemographic characteristics showed a larger population of older adults who were economically disadvantaged, with a significant proportion facing unemployment and limited income, whilst a minority were receiving social welfare support. Poorer self-reported health was associated with older age. The finding aligns with existing literature suggesting that older and unemployed individuals report poorer health, as the likelihood of developing chronic conditions and experiencing functional decline increases, which can negatively impact self-reported health (Petra et al., 2019; Venegas-Sanabria et al., 2023; Dramé et al., 2023). Additionally, individuals with lower socioeconomic status, measured by factors like income, education, and occupation, tend to report poorer self-reported health (Vo et al., 2023; Gallagher et al., 2016). A significant difference was observed in the employment status across different self-reported health groups, with those in “very good” health more likely to be currently working, compared to those not working who reported “not good” self-reported health. This association underscores the impact of health on employment ability; poorer health limits work capacity and opportunities, reinforcing the cycle of health decline and reduced economic activity (Bambra and Eikemo, 2009). Like other studies, the current study showed that adults who reported “not good” health status were more likely to be part of a social welfare program. Some studies suggest that receiving social assistance may be associated with poorer health outcomes (Shahidi et al., 2019; Shao et al., 2022). Self-reported health was not associated with perceived wealth in this sample. However, other studies have demonstrated wealth index to be strongly associated with self-reported health, with wealthier individuals tending to report better health, suggesting that while wealth may not directly impact self-reported health in this dataset, it generally plays a significant role in health outcomes through improved access to essential resources and services such as quality healthcare, nutritious food, safe housing, education, and opportunities for healthy lifestyles (Kyriopoulos et al., 2024; do Amaral Júnior et al., 2023).

The results highlight a complex relationship between self-reported health and functional health dimensions, including psychological and psychosocial factors. Notably, a high proportion of older adults exhibited depressive symptoms in this study, highlighting a substantial mental health burden in this population. Although participants who rated their health as “not good” had slightly higher depression scores than those reporting “very good” health, the difference was not statistically significant, suggesting that self-rated health was not strongly associated with depressive symptom severity in this sample. Depressive symptoms are a significant mental health challenge among older adults in LMICs, with a pooled prevalence of approximately 10.5% and substantial variation across countries, partly due to differences in diagnostic tools and socioeconomic vulnerability (Edwards et al., 2023). Socioeconomic factors such as age, gender, education, poverty, living arrangements, and lower wealth appear to be critical predictors, with limited health safety nets placing older people at increased risk of depression (Brinda et al., 2016). This trend was statistically significant; however, the small number of respondents in the no depressive symptoms category warrants cautious interpretation, as depressive symptoms may not be the only factor influencing self-reported health (Leng et al., 2025). Similarly loneliness, while more prevalent among those with “somewhat” and “not good” self-reported health, did not show significant variation across health categories, suggesting other factors might contribute to feelings of isolation, as evidenced when other factors were adjusted for in the multivariable analysis. Loneliness is a critical health determinant for older adults in LMICs, with research indicating that social isolation, poverty, and physical health challenges are strongly correlated with depression and potentially loneliness (Banerjee et al., 2023). A cross-cultural study found that loneliness can predict mortality across different cultural settings, suggesting its significant health impact (Gao et al., 2021). Older adults reporting higher subjective wellbeing were more likely to report “very good” health compared to those reporting “not good” health status. Self-reported health is strongly associated with subjective wellbeing (Ngamaba et al., 2017). This aligns with the perceived stress scores, where individuals reporting “very good” health status experienced lower stress levels (Axén et al., 2023). Similarly, disability scores and social status perceptions varied across self-reported health categories, with individuals reporting “very good” health having lower disability scores and perceiving themselves to have higher social status. Evidence reveals substantial variation in disability prevalence among older people across LMICs (Lestari et al., 2019). A comprehensive study of a large sample of 53,447 adults from 43 LMIC countries found that 33.3% of older adults reported disability (functional difficulties in affect, cognition, interpersonal activities, mobility, pain and discomfort, self-care, sleep and energy, and vision) with higher rates among females and increasing with age (Hosseinpoor et al., 2016). The significant association between health and life satisfaction illustrates that self-perceived quality of life has far-reaching impacts on an individual’s emotional and social wellbeing (Ngamaba et al., 2017). More than 60% reported “hard” to “very hard” financial strain, along with perceived ill-treatment occurring predominantly outside the home; this points to broader societal challenges affecting the health and wellbeing of older adults. Financial hardship acts as a predictor of health outcomes. Results from five developing countries reveal that financial stress was inversely associated with good self-reported health, with older adults experiencing lower odds of reporting good health under financial strain (Huang et al., 2020). Though most studies are cross-sectional, further longitudinal research is required to establish definitive causal mechanisms. In addition, results from eight countries reveal that older people abuse is recognized as a significant issue in LMICs, with perceptions of abuse categorized into neglect, including isolation and social exclusion, violation of human, legal, and medical rights, and deprivation of choices, decisions, status, and respect (Kalache, 2002; Wamara et al., 2021). These results stress the importance of addressing social factors to improve overall health outcomes and quality of life among older persons.

Hand grip strength is a well-established indicator of overall physical function and can be reflective of broader health status and even mortality risk (Bohannon, 2015). The distribution of hand grip strength across different categories in this study further supports this trend. Notably, higher scores in hand grip strength were more common among individuals reporting “very good” health, whereas lower scores were more prevalent among those with “not good” self-reported health. This pattern is consistent with previous studies that have demonstrated a relationship between physical strength and health outcomes (Rantanen et al., 2012; Ashdown-Franks et al., 2019). In a large study involving 34,129 individuals aged 50 years and above in six LMICs, 47.4% were found to have weak hand grip strength. Weakened grip strength was linked to 1.45 times higher odds of depression (Ashdown-Franks et al., 2019) and an increased likelihood of chronic physical conditions (Firth et al., 2019). Socioeconomic differences were also evident, with higher wealth and better nutritional status showing positive correlations with grip strength (Arokiasamy et al., 2021). Older adults in this study who reported poorer health were more likely to experience higher blood pressure, with a notable increase in Stage 2 hypertension and hypertensive crisis. Studies show a significant association between self-reported poor health status and a higher likelihood of having high blood pressure (hypertension) (Ma et al., 2015). A significant majority, 117 (57.6%), were in the high health risk category based on waist-to-hip ratio, suggesting unfavorable body fat distribution that is associated with increased health risks. Although BMI did not show significant differences across self-reported health categories in this study, it remains a useful measure for identifying at-risk populations and should be considered alongside other indicators of health (Zierle-Ghosh and Jan, 2024; National Academies of Sciences E et al., 2023).

## Conclusion

This study highlights a complex relationship between self-reported health, sociodemographic characteristics, functional measures, and objective health indicators. Key factors linked to self-reported health include age and subjective wellbeing; increasing age was associated with a decline in self-reported health, indicating that older individuals perceive their health less favorably. Conversely, higher levels of subjective wellbeing were strongly associated with better self-reported health, emphasizing the significant role of psychosocial and emotional factors in health perception. Additionally, objective health indicators like male hand grip strength and blood pressure correlated with self-reported health, reinforcing the importance of physical factors in health assessments. Although BMI showed no relationship with self-reported health, it remains useful for identifying older adults at risk for adverse health outcomes. Further research using large longitudinal studies that include contextual factors is needed to clarify some findings. These data inform the development of the full-scale nationally representative LOSHAK, highlighting the importance of collecting sociodemographic, self-reported health, functional, and objective health data to enable deeper analysis of these relationships and explore the potential of self-reported health as an alternative measure of objective health in aging populations in LMIC settings.

## Limitation

One of the limitations of this study was the small sample size, which may affect the generalizability of the findings. To enhance the robustness and applicability of future research, it is recommended that similar studies be conducted with larger and more diverse samples. This is being implemented in the planned national LOSHAK to allow for more comprehensive analysis and stronger conclusions.

## Data Availability

The raw data supporting the conclusions of this article will be made available by the authors, without undue reservation.

## Abbreviations

BMI, Body Mass Index; DBP, Diastolic BP; LOSHAK, Longitudinal Study of Health and Aging in Kenya; LMICs, Low-and-Middle-Income-Countries; SBP, Systolic BP.
